# Using double-bundle Achilles tendon–bone allograft for reconstruction of compound loss of lateral malleolus and lateral ankle ligaments: A rare case report

**DOI:** 10.1097/MD.0000000000051359

**Published:** 2026-11-06

**Authors:** Hsuan Chiu, Jen Hung Chen

**Affiliations:** a Department of Orthopaedic Surgery, Kaohsiung Chang Gung Memorial Hospital, Kaohsiung, Taiwan.

**Keywords:** bone allograft, case report, compound tissue loss, lateral malleolar defect, tendon

## Abstract

**Rationale:**

Composite defects of the lateral ankle, involving bone loss, ligament disruption, and soft tissue compromise, cause profound mechanical instability and are difficult to reconstruct with techniques that address bone or ligaments alone.

**Patient concerns:**

We report a 26-year-old woman who presented with progressive right ankle pain, recurrent giving-way, and peroneal tendon subluxation 7 years after a high-energy crush injury that required flap coverage.

**Diagnoses:**

Lateral malleolar loss with a distal fibular bony defect measuring approximately 5 × 3 × 2 cm and chronic varus instability; complete anterior talofibular ligament (ATFL) and calcaneofibular ligament (CFL) rupture; and peroneal tendon dislocation were confirmed by stress radiographs and magnetic resonance imaging.

**Interventions:**

Single-stage reconstruction with a cryopreserved Achilles tendon–bone allograft. The calcaneal bone block was contoured and fixed to the fibular stump with a 4.5-mm cannulated screw to reconstitute the lateral malleolus. The tendon was split into 2 bundles (7 mm × 30 mm for the ATFL and 7 mm × 40 mm for the CFL) for anatomic ATFL and CFL reconstruction and secured with interference screws at the talar and calcaneal footprints. The peroneal tendons were reduced after bony restoration. Postoperative rehabilitation used an Aircast^®^ brace with non-weight-bearing ambulation for the first 3 weeks, partial weight-bearing from week 3, full weight-bearing by week 6, and brace discontinuation at week 12.

**Outcomes:**

At the final follow-up of 16 months, the patient ambulated pain-free with a stable ankle. Radiographs demonstrated successful incorporation of the bone allograft, and magnetic resonance imaging confirmed intact ATFL/CFL grafts with maintained peroneal tendon reduction. Functional scores were Karlsson–Peterson 87 and American Orthopaedic Foot & Ankle Society ankle–hindfoot 90.

**Lessons:**

A double-bundle Achilles tendon–bone allograft enables simultaneous reconstruction of lateral malleolar bone and lateral ligaments, avoiding donor-site morbidity and achieving durable stability in complex, previously reconstructed ankles. This approach should be considered when both osseous and ligamentous deficits coexist.

## 1. Introduction

Open ankle fractures are relatively uncommon and are most often associated with high-energy trauma, such as motor vehicle collisions or crush injuries.^[1]^ In severe cases, these injuries may result in a composite loss that includes the lateral malleolus, lateral ankle ligaments – particularly the anterior talofibular ligament (ATFL) and calcaneofibular ligament (CFL) – and the overlying soft tissue envelope. Such complex injuries severely compromise the structural integrity and biomechanical stability of the ankle joint, frequently leading to talar lateralization, varus instability, and an increased risk of posttraumatic osteoarthritis if not adequately reconstructed.^[1,2]^

The surgical management of these composite defects presents significant challenges, as it requires the simultaneous restoration of bony architecture, ligamentous function, and soft tissue coverage. Several techniques have been described for reconstructing lateral malleolar defects, including autologous iliac crest bone grafting, patellar tendon–bone grafts, and fibular head transfers.^[3–8]^ While these approaches can provide structural replacement, they are often associated with considerable donor-site morbidity and, more importantly, fail to address the concurrent deficiency of the lateral ankle ligaments. This limitation can result in persistent instability and suboptimal long-term functional outcomes.

In particular, the ATFL and CFL play a critical role in providing lateral ankle stability and proprioceptive feedback during gait and athletic activity. Therefore, comprehensive reconstruction of these ligaments is essential in achieving functional restoration. Traditional techniques may be inadequate in the context of extensive bone loss or previously compromised soft tissue, such as in patients with prior flap coverage or chronic wounds. To address these multifaceted challenges, we present a novel and integrative surgical technique utilizing a double-bundle Achilles tendon–bone allograft for concurrent reconstruction of the lateral malleolus, ATFL, and CFL. This approach restores both the anatomical framework and the mechanical function of the lateral ankle stabilizers.

## 2. Case presentation

This case report was exempt from institutional review board approval per institutional policy, as it involved a retrospective description of routine clinical care without additional intervention. Written informed consent was obtained from the patient for publication of this case report and accompanying images.

A 26-year-old female presented with progressive right ankle discomfort and recurrent episodes of instability during ambulation. These symptoms had worsened over the previous 1.5 months, following a minor vehicular accident. The patient was a sedentary office worker with a low level of physical activity (height 158.5 cm, weight 54 kg; body mass index of 21.5 kg/m^2^) and a nonsmoker. Her medical history was significant for a major motorcycle-related crush injury sustained 7 years prior, resulting in a large composite defect (15 × 8 cm) involving the skin, subcutaneous tissue, and exposure of bone and tendons along the lateral aspect of the right ankle (Fig. 1). The radiograph obtained at the time of this index injury confirms that the distal fibular bone loss was already present as a direct result of the initial crush trauma, rather than developing as a complication of the subsequent primary reconstruction. The initial injury had been treated with a free anterolateral thigh flap procedure, which addressed the soft tissue defect but did not include bony or ligamentous reconstruction of the lateral malleolus, accounting for the persistent bone and ligament deficiency seen at the current presentation. Over the subsequent years, the patient experienced intermittent sensations of lateral ankle instability and subluxation of the tendons, though these were not functionally limiting until recently. On physical examination, she exhibited reduced ankle mobility and marked varus laxity. There was no notable swelling, ecchymosis, or signs of infection. Plain radiographs showed segmental loss of the distal fibula, measuring approximately 5 × 3 × 2 cm. Varus stress radiographs demonstrated a talar tilt angle of 13.3°, with no joint space widening or anterior talar translation on the ankle anteroposterior and lateral views (Fig. 2). Magnetic resonance imaging revealed complete rupture of the ATFL and CFL, peroneal tendon dislocation with a torn superior peroneal retinaculum and a flat fibular groove morphology, without obvious cartilage thinning or erosion (Fig. 3). The previously reconstructed soft tissue flap was intact and viable. Given the complex nature of the injury – including osseous, ligamentous, and soft tissue defects – a single-stage reconstruction was undertaken. The goals were anatomical restoration of ankle stability, preservation of existing soft tissue coverage, and prevention of long-term joint degeneration.

**Figure 1.**
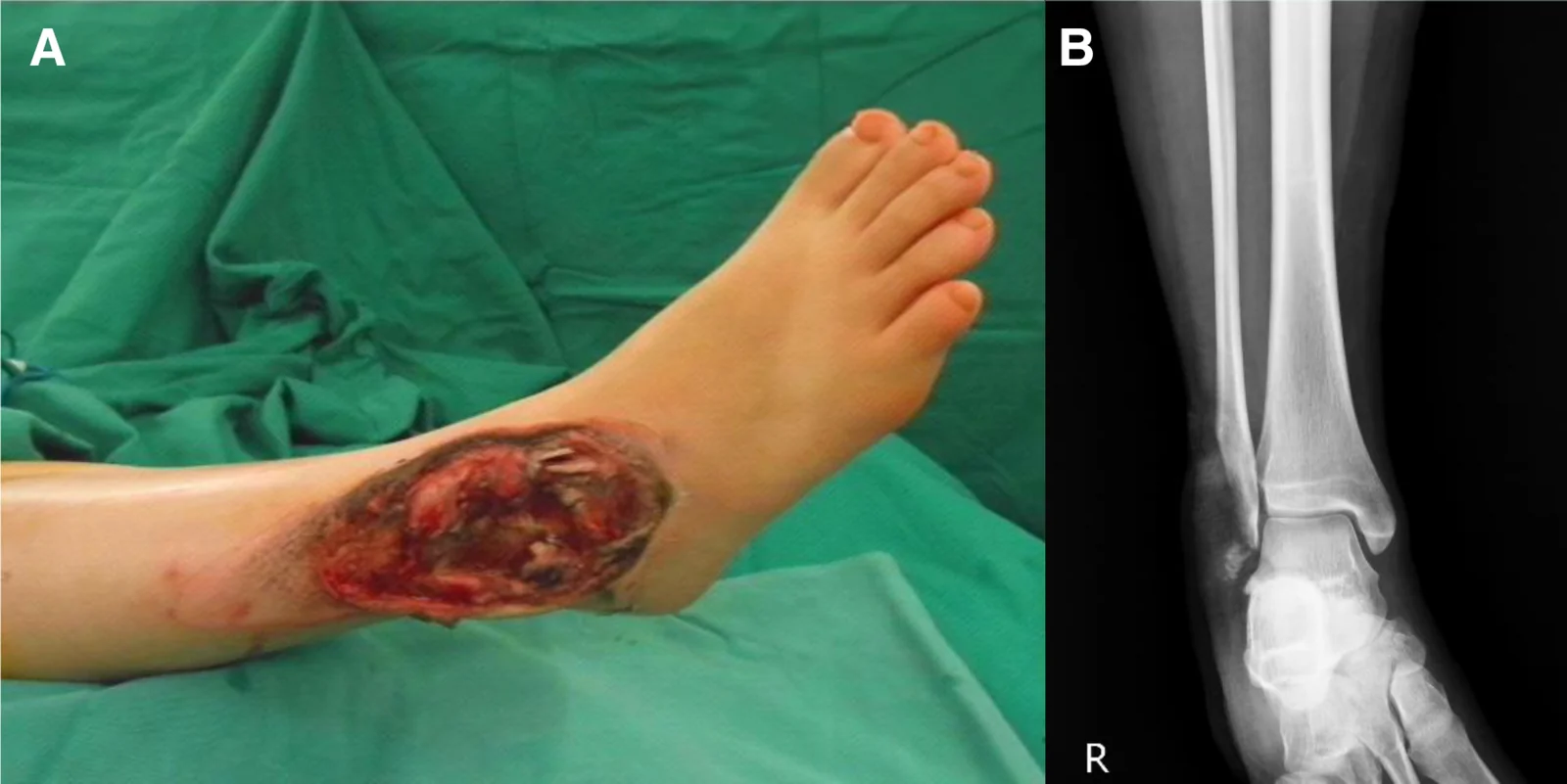
(A) Preoperative clinical photograph taken at the time of injury, 7 years prior, demonstrating a large composite soft tissue defect with exposure of bone and tendon over the lateral aspect of the right ankle. (B) Plain radiograph of the right ankle taken at the time of injury demonstrated a bony defect of the lateral malleolus.

**Figure 2.**
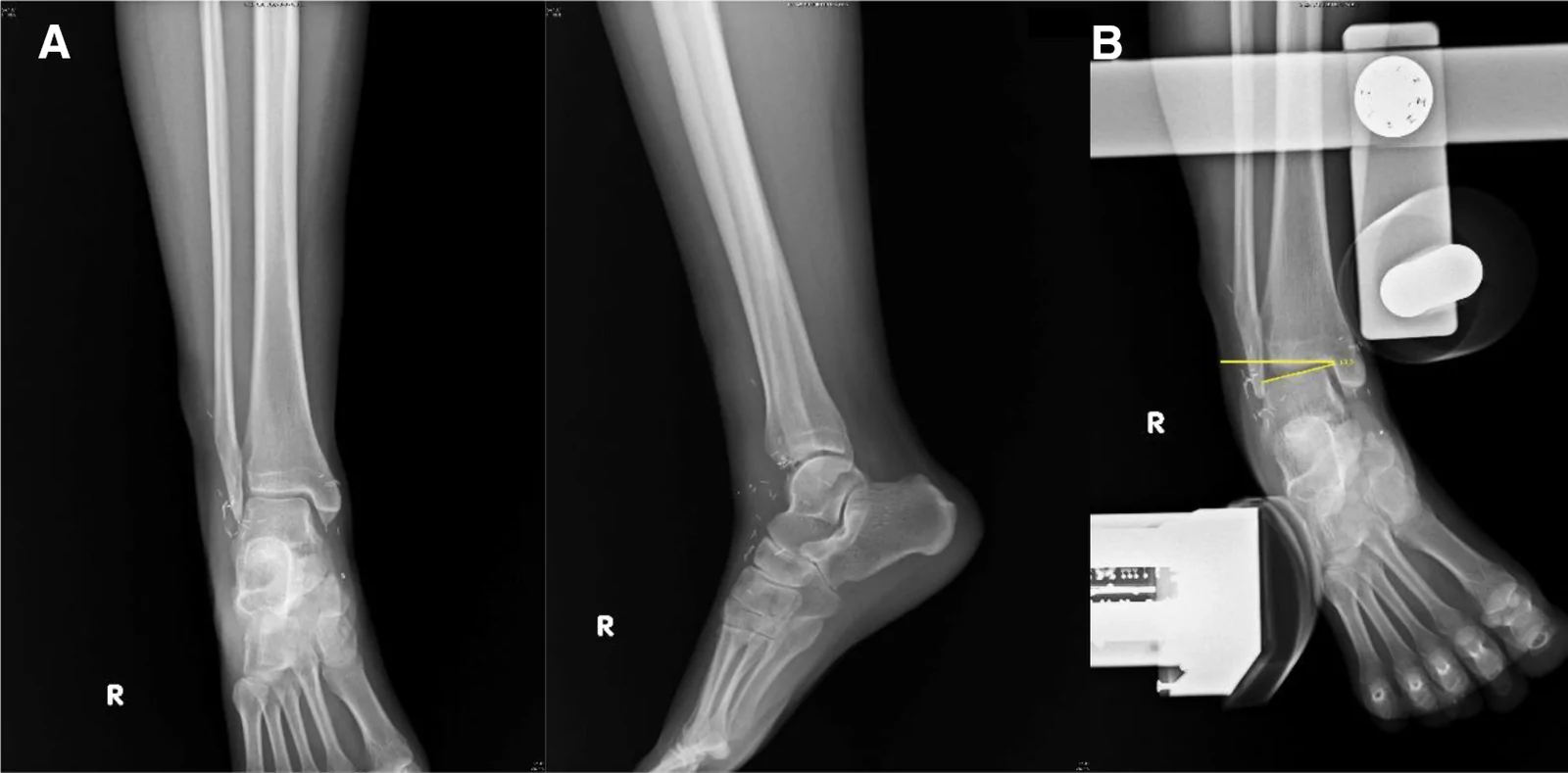
(A) Preoperative plain radiographs of the right ankle showed segmental loss of the distal fibula, measuring approximately 5 × 3 × 2 cm. (B) Varus stress view demonstrating a talar tilt angle of 13.3°, with no joint space widening or anterior talar translation on the anteroposterior and lateral views, consistent with chronic lateral ligament insufficiency and varus instability.

**Figure 3.**
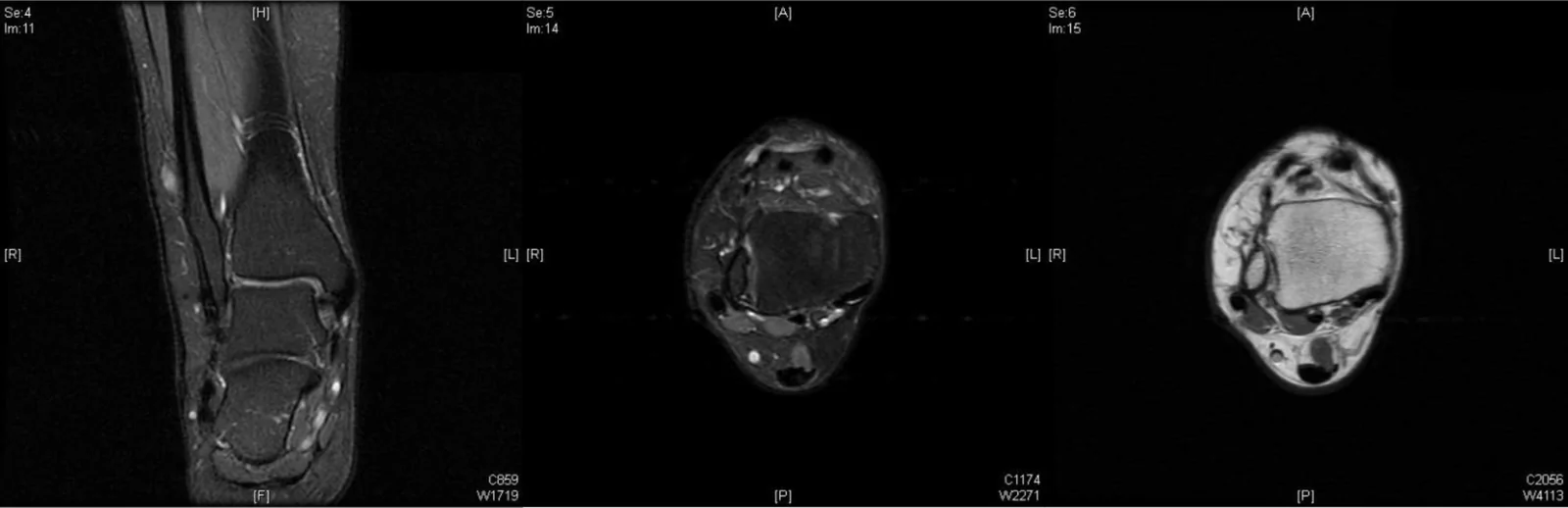
Preoperative magnetic resonance imaging reveals a complete rupture of the anterior talofibular ligament and calcaneofibular ligament, accompanied by dislocation of the peroneal tendons with a torn superior peroneal retinaculum and a flat fibular groove morphology, without obvious cartilage thinning or erosion.

The surgical procedure was performed under general anesthesia with the patient in a supine position. A surgical approach of approximately 14 cm was made through the posterior margin of the previously reconstructed flap (Fig. 4). Preoperative imaging was used to define the size of the lateral malleolar defect. A cryopreserved, nonirradiated Achilles allograft with a calcaneal bone block (FlexiGraft® Achilles; LifeNet Health) was selected. The calcaneal bone segment of the allograft was contoured using an oscillating saw and rongeur to fill the bony gap. The tendon segment was split longitudinally into 2 bundles: 1 measuring 7 mm × 30 mm for the ATFL and another 7 mm × 40 mm for the CFL. Both graft ends were whip-stitched using No. 2 Ethibond sutures (Ethicon, Johnson & Johnson; Fig. 5). The contoured calcaneal bone block was fixed to the remaining fibular stump using a 4.5-mm cannulated screw and washer over a 3.2-mm drill hole, under fluoroscopic guidance. Bone sockets 30 mm in depth were prepared with a 5.0-mm drill at the anatomical attachment sites – a talar socket for the ATFL limb and a calcaneal socket for the CFL limb. Each graft limb was pulled taut on its whip-stitch sutures and firmly fixed within its respective socket using a 5.0 × 25 mm bioabsorbable interference screw (Arthrex) with the ankle positioned neutrally. Fibular length and rotational alignment were confirmed intraoperatively under fluoroscopic guidance. Balanced tension of the ATFL and CFL bundles was confirmed intraoperatively by taking the ankle through a full passive range of motion: the ATFL bundle tightened in plantarflexion and relaxed in dorsiflexion, while the CFL bundle showed the reciprocal pattern, tightening in dorsiflexion and relaxing in plantarflexion. Anterior drawer testing in slight plantarflexion and talar tilt testing with hindfoot inversion demonstrated a firm endpoint without excessive translation or tilt, and subtalar range of motion remained unrestricted, confirming that the CFL bundle was not overtensioned. Intraoperative stress testing of the tibiofibular syndesmosis demonstrated stability, and a syndesmotic screw was therefore not used. Following bony reconstruction, the peroneal tendons were found to have spontaneously reduced (Figs. 6 and 7).

**Figure 4.**
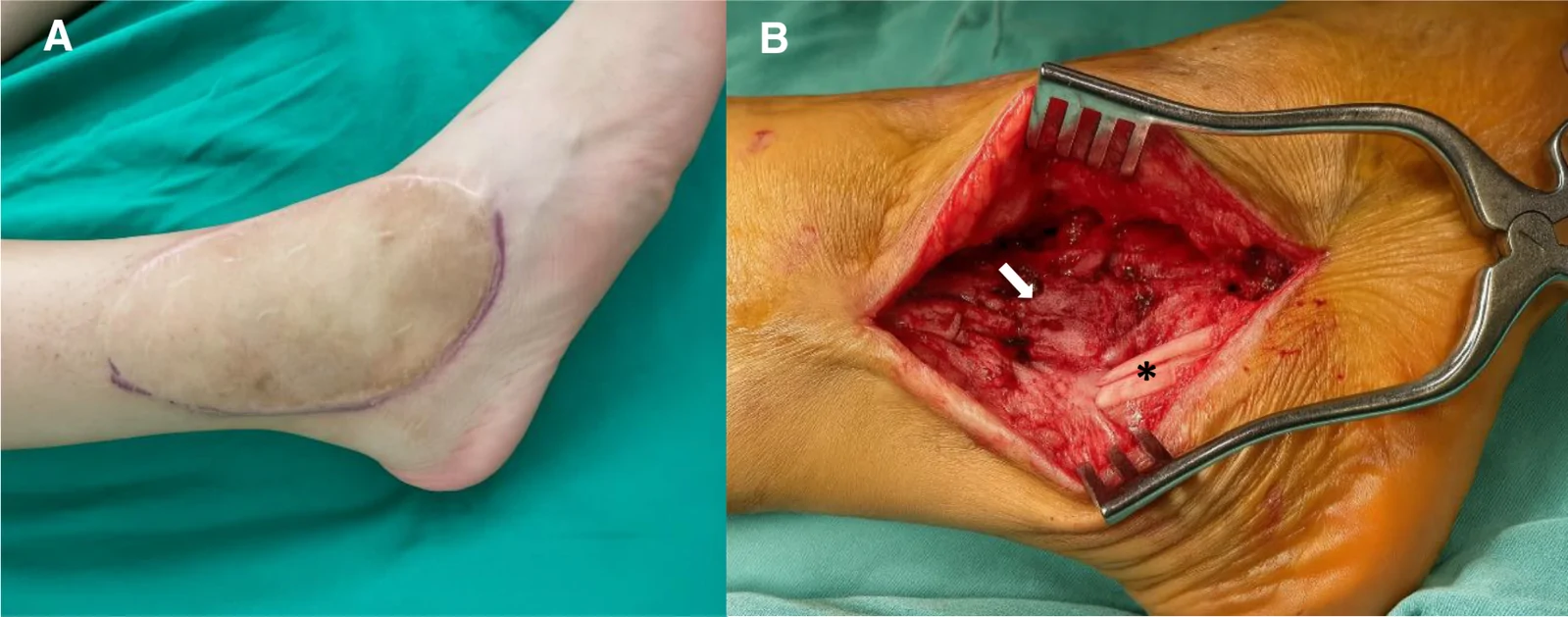
Intraoperative views of the right ankle. (A) Preoperative photograph showing the healed skin flap from prior reconstruction. (B) Intraoperative exposure revealing compound loss of the lateral malleolus (arrow), lateral ankle ligaments, and dislocation of the peroneus brevis and longus tendons (asterisk).

**Figure 5.**
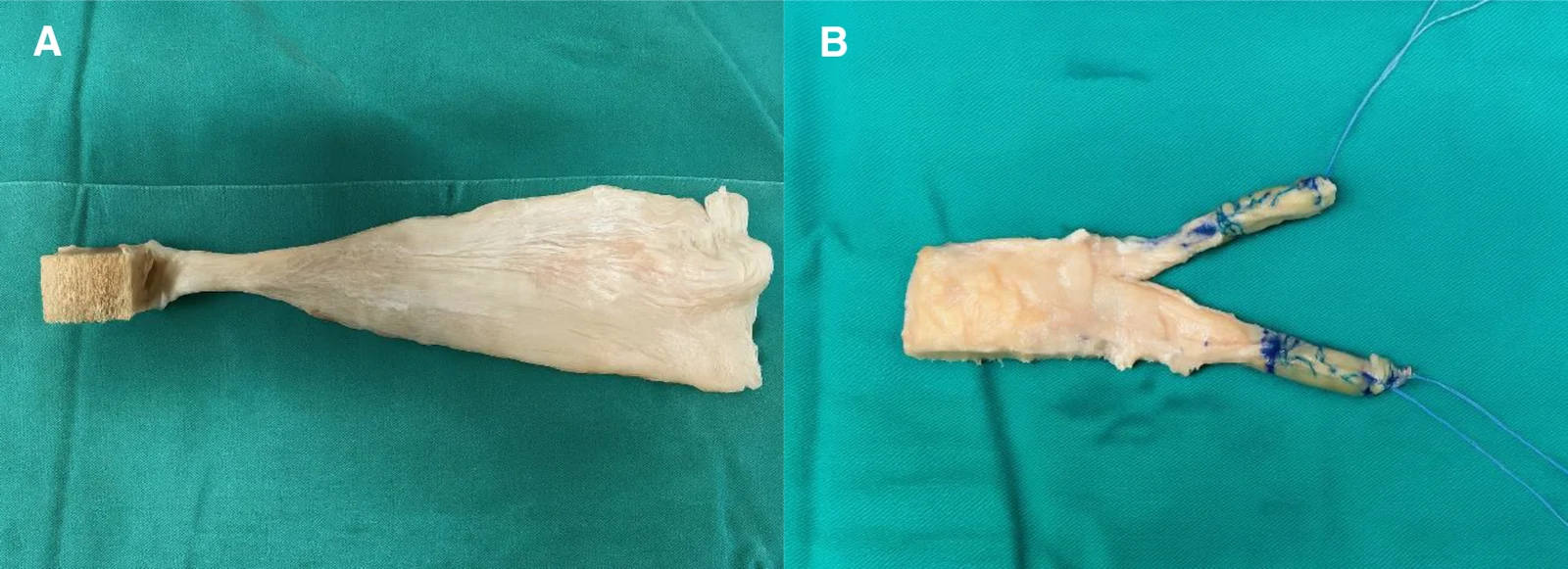
Preparation of Achilles tendon allograft with a calcaneal bone block. (A) The intact Achilles allograft (FlexiGraft^®^) with the calcaneal bone segment prior to modification. (B) The calcaneal bone block was contoured to fill the fibular gap. The tendon portion was longitudinally split into 2 bundles to reconstruct the anterior talofibular ligament and calcaneofibular ligament. Both graft ends were whip-stitched using No. 2 Ethibond sutures.

**Figure 6.**
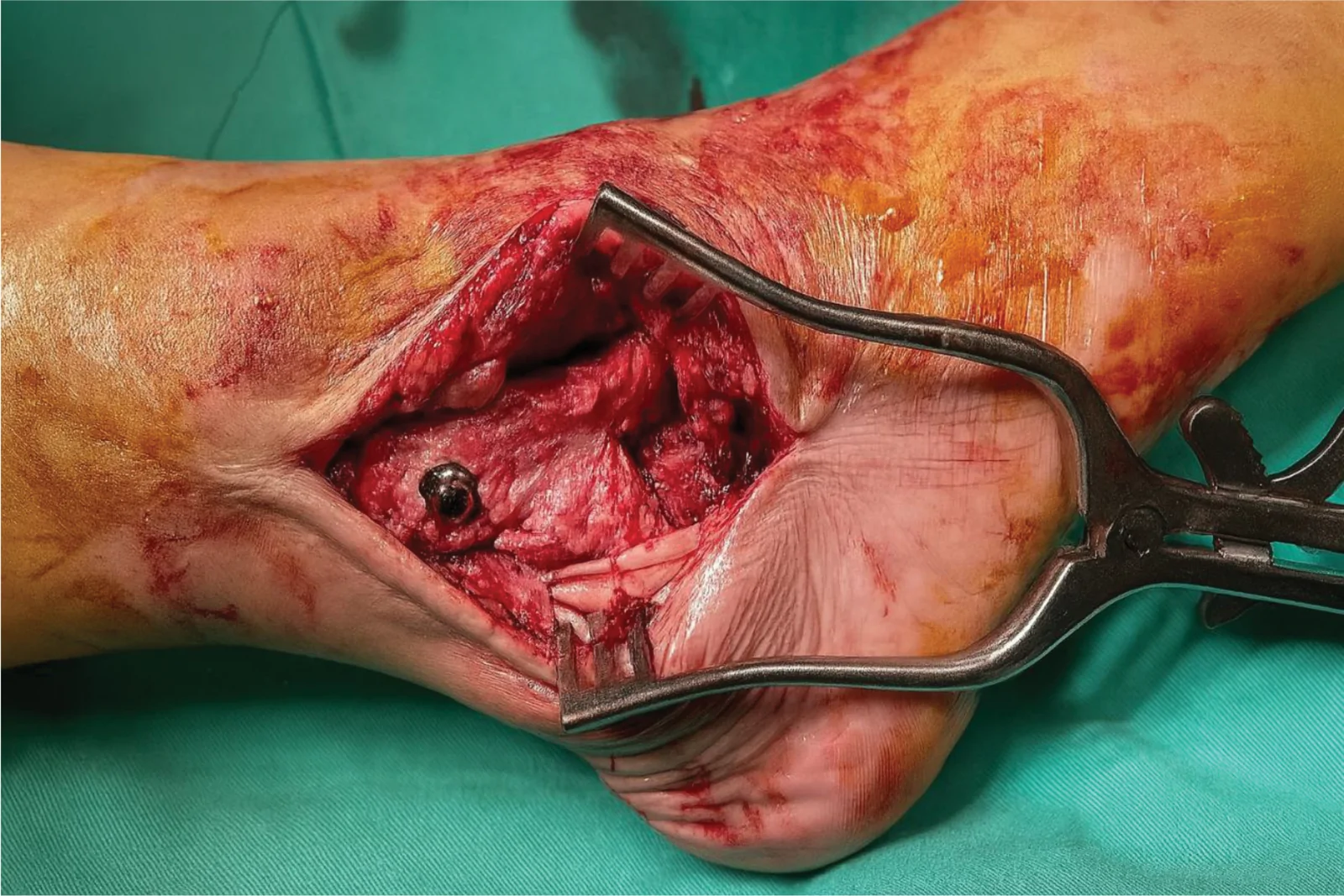
Reconstruction of the lateral malleolus, anterior talofibular ligament, and calcaneofibular ligament using a double-bundle Achilles tendon–bone allograft.

**Figure 7.**
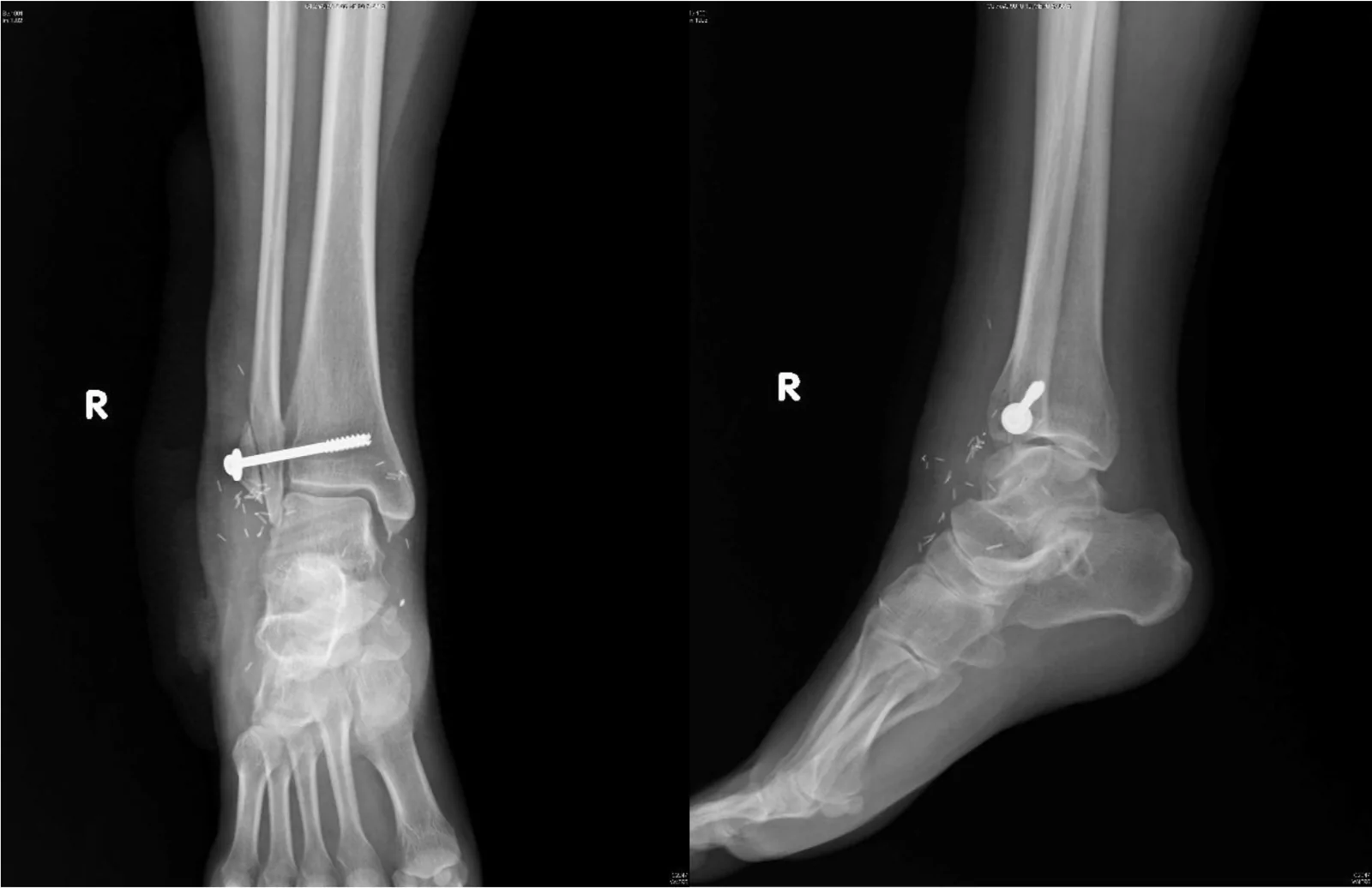
Postoperative radiographs of the right ankle demonstrating fixation of the contoured calcaneal bone block to the distal fibula using a 4.5-mm cannulated screw with a washer.

An Aircast® brace was applied on postoperative day 1, with a non-weight-bearing ambulation program for the first 3 weeks. Partial weight-bearing was permitted from postoperative week 3 while continuing Aircast® brace use, with progression to full weight-bearing by postoperative week 6. The brace was discontinued at approximately postoperative week 12. Weight-bearing lateral malleolar pain, present early in the postoperative course, had resolved by 6 months. At the final follow-up of 16 months, the patient had regained full independent ambulation without pain. Clinical examination confirmed stable lateral ankle structures and unrestricted range of motion with no residual laxity. Follow-up radiographs demonstrated partial resorption of the fibular bone allograft; screw fixation remained stable throughout follow-up without evidence of hardware backing out (Fig. 8). The patient reported no hardware irritation, and implant removal was therefore not planned. Magnetic resonance imaging demonstrated intact ATFL and CFL reconstructions with no evidence of graft retear, with maintained reduction of the previously dislocated peroneal tendons (Fig. 9). The final functional assessments revealed scores of 87 on the Karlsson–Peterson ankle instability scale and 90 on the American Orthopaedic Foot & Ankle Society ankle–hindfoot scale, indicating an excellent clinical outcome. The overall clinical course, from the index injury through final follow-up, is summarized in the timeline in Figure 10.

**Figure 8.**
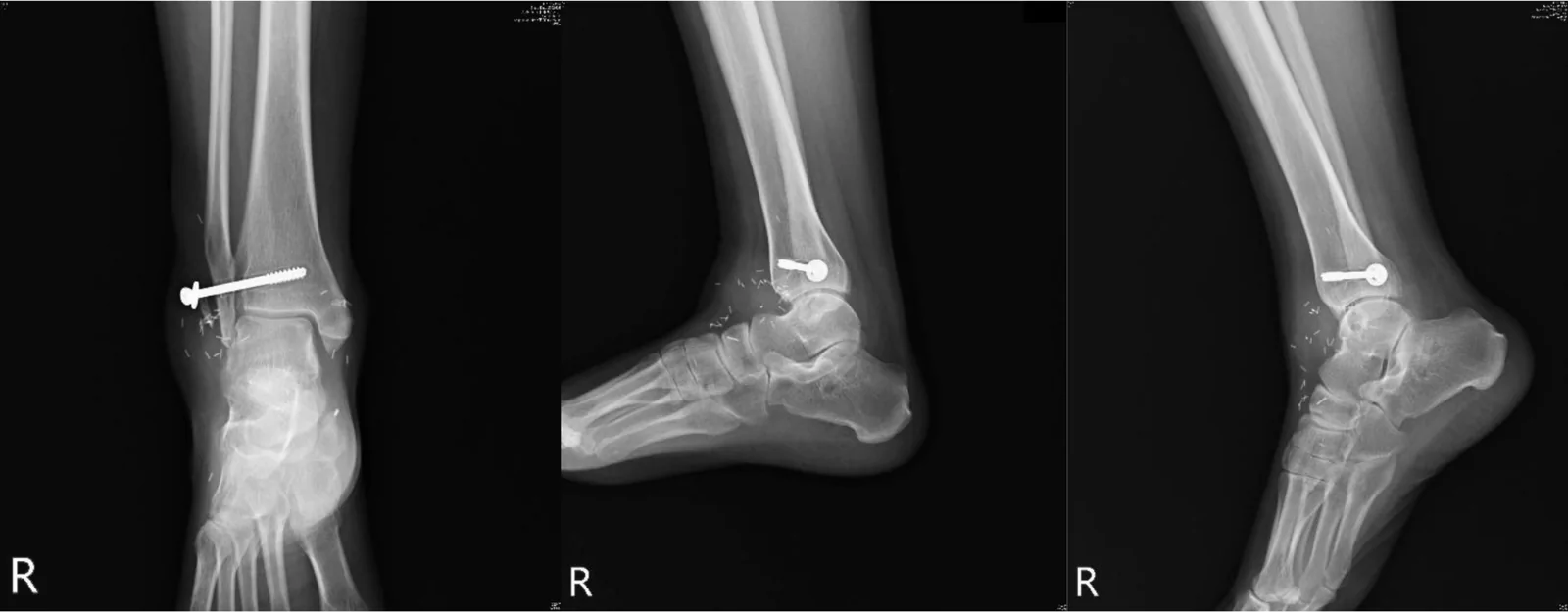
Follow-up radiographs of the right ankle at 16 months postoperatively showed successful incorporation of the calcaneal bone block into the distal fibula with maintained screw fixation.

**Figure 9.**
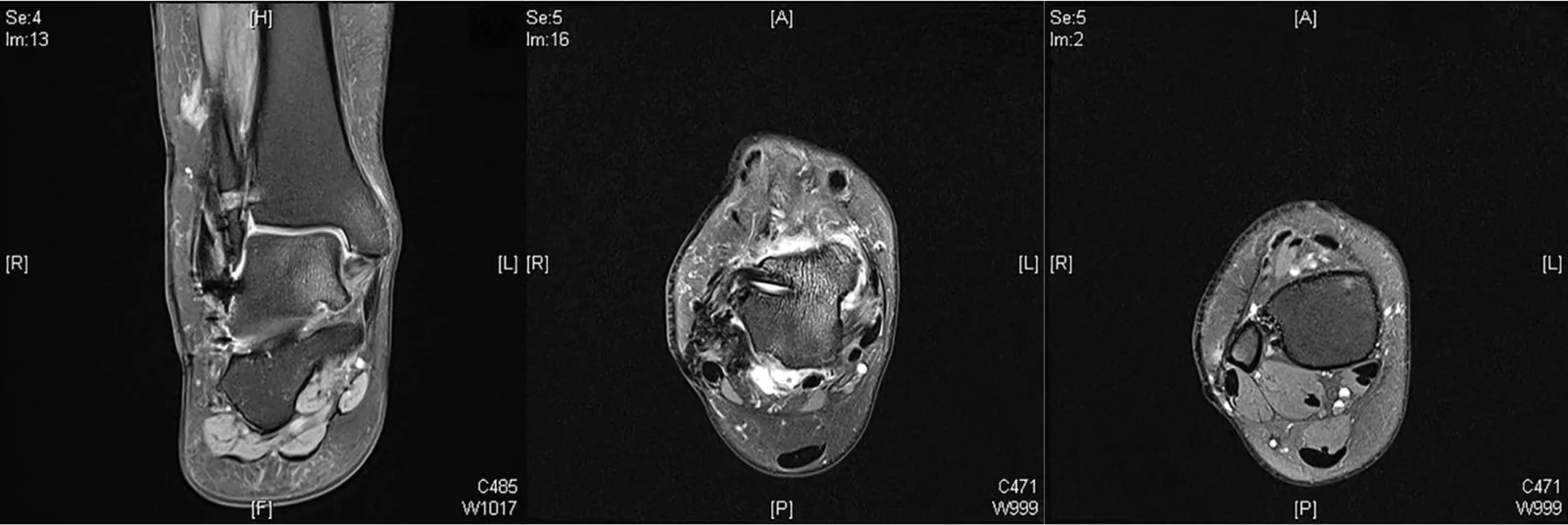
16-month postoperative magnetic resonance imaging of the right ankle. Coronal view shows intact anterior talofibular ligament and calcaneofibular ligament reconstructions. Axial view confirms maintained reduction of the previously dislocated peroneal tendons.

**Figure 10.**
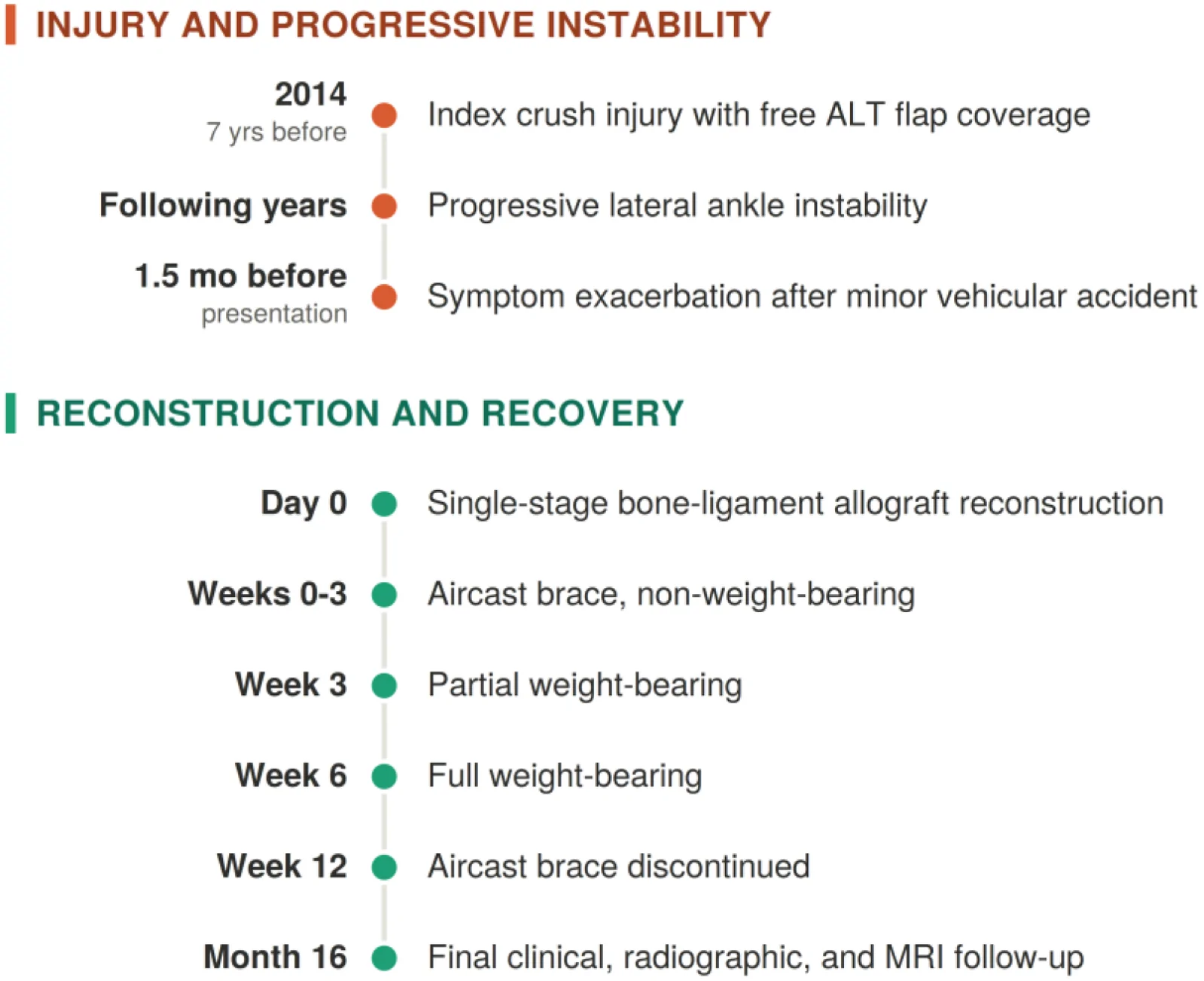
Timeline of the 7-year clinical course, from the index crush injury and free ALT flap coverage through progressive lateral instability, single-stage bone–ligament allograft reconstruction, the postoperative rehabilitation protocol, and final follow-up at 16 months. ALT = anterolateral thigh.

## 3. Discussion

Open fractures involving the ankle, although infrequent, are often the result of high-impact trauma such as crush injuries or vehicular accidents.^[1]^ When these injuries involve extensive loss of the lateral malleolus, lateral ankle ligaments, and surrounding soft tissue, they present a complex reconstructive challenge. Without appropriate restoration of bony and ligamentous structures, the resulting instability may lead to lateral talar displacement and eventual posttraumatic arthropathy.^[1,2]^

Effective treatment of such composite injuries requires a multidimensional approach that simultaneously addresses skeletal support, ligament function, and soft tissue coverage. Previous reports have proposed techniques such as iliac crest bone grafting^[3,4]^ and vascularized fibular head transfer to manage distal fibular defects.^[5–8]^ While these methods provide osseous continuity, they carry risks of donor-site complications and do not inherently resolve the issue of lateral ligament insufficiency.

In some cases, joint-sacrificing procedures such as ankle arthrodesis have been used, as described by Behera et al, to alleviate pain and manage instability in patients with lateral malleolar loss.^[9]^ However, fusion compromises joint mobility and may not be ideal in younger individuals seeking to preserve function. Alternatively, Cheng et al introduced the use of 3D-printed titanium prostheses for distal fibula reconstruction, offering structural support but not ligamentous restoration.^[10]^ Wang and Yu also reviewed emerging reconstructive methods, emphasizing the need for integrated techniques that address both bony and ligamentous defects.^[11]^

In our case, we employed a double-bundle Achilles tendon–bone allograft to simultaneously reconstruct the lateral malleolus, ATFL, and CFL. The Achilles tendon–bone allograft was selected because it was the only tendon–bone composite allograft available under our institution tissue bank contract; beyond availability, it was also anatomically and biomechanically well-suited to this reconstruction, as its robust tensile strength and generous tendon length allowed the graft to be split into 2 independent bundles of adequate size for both the ATFL and CFL, while its attached calcaneal bone block provided structural bone stock matched in size and contour to the fibular defect, enabling bone and ligament reconstruction with a single, biologically integrated implant. This strategy allowed for both anatomical and biomechanical restoration of the lateral ankle complex. The ATFL and CFL are essential for maintaining lateral and rotational stability of the ankle, and their anatomical reconstruction has been shown to be crucial for achieving favorable long-term results.^[12–14]^

Conventional ligament reconstruction techniques, including free tendon grafting, are widely accepted for isolated chronic lateral ankle instability.^[15–18]^ However, these procedures are not well-suited for cases involving segmental bone loss. The combined bone–tendon allograft used in our patient allowed secure fixation and replication of the anatomical footprint of both ligaments, restoring stability in a biologically and mechanically integrated manner.

The use of allograft tissue introduces considerations such as the risk of immunogenic response, disease transmission, and delayed biological incorporation.^[19]^ In our case, meticulous debridement, thorough irrigation, and early soft tissue coverage contributed to uneventful graft healing. Soft tissue coverage was maintained by the patient preexisting free anterolateral thigh flap from the original reconstruction, which remained viable and intact throughout the current procedure and required no additional flap coverage.

This report has several limitations. As a single-case study, our findings demonstrate technical feasibility rather than proof of efficacy or superiority over existing reconstructive options. Baseline functional scores prior to the index procedure were not available for comparison, as instability had been present for years without formal outcome assessment. No comparator technique or control group was included. Finally, the 16-month follow-up, while demonstrating early graft incorporation and functional stability, is insufficient to assess long-term allograft durability, the risk of delayed ligament attenuation, or the development of posttraumatic osteoarthritis, which typically manifests over a longer time horizon. In addition, although no hardware complications or nonunion occurred in this case, partial graft resorption was noted radiographically, underscoring the need for continued monitoring of graft incorporation and hardware stability in future cases.

To date, there are limited reports on the use of a double-bundle Achilles tendon–bone allograft for simultaneous reconstruction of both bone and ligament components in the lateral ankle. Our case demonstrated excellent short-term functional recovery and joint stability without the morbidity associated with autograft harvest or microsurgical procedures.

## 4. Conclusion

In this single case, a double-bundle Achilles tendon–bone allograft achieved technically feasible, single-stage reconstruction of a complex lateral ankle defect involving combined bone and ligament loss, with a favorable functional and radiographic outcome at 16 months. While these findings suggest this technique may be a useful option in similarly complex cases with chronic instability and prior soft tissue compromise, further studies with larger cohorts and longer follow-up are needed to establish its efficacy, superiority over existing techniques, and long-term durability.

## Acknowledgments

The authors thank the patient for granting permission to use clinical images and case details for publication.

## Author contributions

**Data curation:** Hsuan Chiu, Jen Hung Chen.

**Conceptualization:** Jen Hung Chen.

**Investigation:** Jen Hung Chen.

**Methodology:** Jen Hung Chen.

**Project administration:** Jen Hung Chen.

**Supervision:** Jen Hung Chen.

**Writing – original draft:** Hsuan Chiu.

**Writing – review & editing:** Jen Hung Chen.
